# Effects of Small Intestinal Submucosa (SIS) on the Murine Innate Immune Microenvironment Induced by Heat-Killed *Staphylococcus aureus*

**DOI:** 10.1371/journal.pone.0048724

**Published:** 2012-11-26

**Authors:** Roshni Roy Chowdhury, Youssef Aachoui, Swapan K. Ghosh

**Affiliations:** Department of Biology, Indiana State University, Terre Haute, Indiana, United States of America; Charité-University Medicine Berlin, Germany

## Abstract

The use of biological scaffold materials for wound healing and tissue remodeling has profoundly impacted regenerative medicine and tissue engineering. The porcine-derived small intestinal submucosa (SIS) is a licensed bioscaffold material regularly used in wound and tissue repair, often in contaminated surgical fields. Complications and failures due to infection of this biomaterial have therefore been a major concern and challenge. SIS can be colonized and infected by wound-associated bacteria, particularly *Staphylococcus aureus*. In order to address this concern and develop novel intervention strategies, the immune microenvironment orchestrated by the combined action of *S. aureus* and SIS should be critically evaluated. Since the outcome of tissue remodeling is largely controlled by the local immune microenvironment, we assessed the innate immune profile in terms of cytokine/chemokine microenvironment and inflammasome-responsive genes. BALB/c mice were injected intra-peritoneally with heat-killed *S. aureus* in the presence or absence of SIS. Analyses of cytokines, chemokines and microarray profiling of inflammasome-related genes were done using peritoneal lavages collected 24 hours after injection. Results showed that unlike SIS, the *S. aureus*-SIS interactome was characterized by a Th1-biased immune profile with increased expressions of IFN-γ, IL-12 and decreased expressions of IL-4, IL-13, IL-33 and IL-6. Such modulation of the Th1/Th2 axis can greatly facilitate graft rejections. The *S. aureus*-SIS exposure also augmented the expressions of pro-inflammatory cytokines like IL-1β, Tnf-α, CD30L, Eotaxin and Fractalkine. This heightened inflammatory response caused by *S. aureus* contamination could enormously affect the biocompatibility of SIS. However, the mRNA expressions of many inflammasome-related genes like Nlrp3, Aim2, Card6 and Pycard were down-regulated by heat-killed *S. aureus* with or without SIS. In summary, our study explored the innate immune microenvironment induced by the combined exposure of SIS and *S. aureus*. These results have practical implications in developing strategies to contain infection and promote successful tissue repair.

## Introduction

The porcine-derived small intestinal submucosa is a clinically approved surgical scaffold routinely used as a biological tissue support for wound healing and tissue remodeling [1]–[4]. SIS is essentially an organic biomaterial consisting primarily of collagen (Types I, III and V) [5]–[7], a constituent of the extracellular matrix (ECM). Other ECM components like glycosaminoglycans, proteoglycans, fibronectin and b-FGF are present in SIS in small amounts [3], [8], [9]. In spite of its xenogeneic origin, SIS has been successfully used in the management of complex abdominal wall defects [10] and Peyronie's disease [11], as dural substitutes [12], anal fistula plugs [13] and many others. This has been possible since SIS comprises evolutionarily conserved proteins of the ECM (nature's ideal biological scaffold) that do not elicit any vigorous and adverse immune response. Furthermore, SIS is completely degraded and replaced by the host tissue over time [14], [15]. The SIS biomaterial has also been evaluated as a vaccine carrier and adjuvant [16]. It has shown promise as a cancer vaccine adjuvant against human prostate cancer by evoking effective cell-mediated immunity [17]. In an earlier study, we have reported the efficiency of SIS in augmenting the immunogenic potential of soluble protein antigens like ovalbumin and keyhole-limpet hemocyanin [18]. We also described that SIS exhibits adjuvanticity without inducing any adverse pathologic inflammatory response even in autoimmune prone NZB/WF1 mice [19]. In this respect, SIS is better than some commercial adjuvants that often induce inflammatory tissue damage at sites of injection. The particulate nature of SIS may also promote phagocytosis, antigen uptake and retention by antigen presenting cells (APCs) [18].

The efficacy of SIS as a bioscaffold has been largely attributed to a Th2-biased immune response and a mild or non-inflammatory immune profile [20]. Analysis of the Th1/Th2 balance induced by SIS has shown a robust IL-4 (Th2) expression with a characteristic IgG1 antibody response [18], [21]. The Th2 response, characterized by the expression of cytokines like IL-4, IL-10, IL-13, favors transplant acceptance. On the other hand, the hallmark cytokine profile of Th1 response (IFN-γ, IL-12, TNF-α), activates macrophages and complement-fixing antibodies leading to xenogeneic transplant rejection [22]. Analysis of the early inflammatory events is critical in assessing the potential of a bioscaffold material since the innate immune system is known to play a vital role in determining the biocompatibility of biomaterials [23]–[25]. It is also important in the evaluation of adjuvanticity since the initial innate immune microenvironment orchestrates the subsequent development of a robust adaptive immune response. The early innate immune proteome (cytokines, chemokines and growth factors) also shapes the wound healing process by stimulating the influx of inflammatory and connective tissue cells [26]. Alterations in the localized innate immune profile may therefore impact the adjuvant properties, promote transplant rejection and decrease the likelihood of wound healing.

Potential complications at the surgical site due to infection remain a major challenge in wound healing and tissue remodeling. Infection of bioscaffold materials has been a serious problem [27], [28] and studies have reported SIS to be susceptible to bacterial colonization and infection [29], [30]. *Staphylococcus aureus*, a gram positive bacterium, is the primary cause of postsurgical wound infections [31] and therefore a major contaminant of biological scaffolds. It is a versatile and ubiquitous microbe that survives as a commensal on the nose, throat and skin of nearly 50% of the adult population [32]. However, it is also the source of various life-threatening conditions such as endocarditis, meningitis and osteomyelitis [33]. In order to evaluate the initial *S. aureus*-SIS-host interactome without inducing infection, we used heat-killed *S. aureus* (HKSA) since both live and HKSA are known to bind collagen with very high affinities [34]. This ability of *S. aureus* to bind to elements of the ECM with high affinity is an essential attribute of the bacterium's invasive properties. HKSA and SIS were injected intra-peritoneally for two major reasons: first, SIS being derived from the small intestine is routinely used for gastrointestinal healing and cecal wound repair [35], [36] and second, *S. aureus*-induced peritonitis remains a major postoperative complication [37].

In this study, we sought to understand the dynamics of the innate immune profile orchestrated by the combined action of SIS and *S. aureus*. The immune microenvironment induced as a result of infection or presence of inflammatory bacterial products such as endotoxins or lipotechoic acids could determine the success or failure of an implant or surgical manipulations as well as wound healing. We therefore addressed the following: how does SIS, in the presence of *S. aureus*, modulate (a) the Th1/Th2 cytokine profile; (b) the milieu of cytokines, chemokines and growth factors; and (c) consequently the inflammasome-responsive genes? Our results demonstrated a Th1-biased cytokine profile (with increased expression of IFN-γ and IL-12) in the presence of *S. aureus* together with SIS. This clearly represents a shift from the Th2 cytokine profile known to be induced by SIS alone [20], [38]. Previously, we have reported that SIS by itself is mildly inflammatory [18]. Addition of *S. aureus* to SIS was found to alter the expression patterns of a number of inflammatory cytokines, like IL-6, IL-1α, IL-1β, Tnf-α and sTNFRs (I and II), chemokines, like CD30L, Eotaxin, Fractalkine, I-TAC, MIG, TECK, LIX and MCP-1, and growth factors, like M-CSF and GM-CSF. Such alterations in the innate immune profile may have significant consequences on inflammation, wound healing and tissue remodeling. This report provides insights into the innate immune microenvironment induced by the dual action of *S. aureus* and SIS, and will therefore be helpful in developing novel strategies for wound-associated infection containment and successful tissue remodeling.

## Materials and Methods

### Bacterial inoculum preparation

*S. aureus* (ATCC number: 25923), kindly provided by Dr. H. K. Dannelly of the Department of Life Sciences, Indiana State University, was used for all experiments since this strain has been shown to exhibit high-affinity, specific-binding to collagen [34]. *S. aureus* was grown overnight in Luria-Bertani (LB) broth (Difco, Detroit, MI) at 37°C for 14–16 hours and harvested with phosphate buffered saline (PBS). Cells were washed in PBS; the culture concentration was determined by spectrophotometry (OD_600_) and then suspended to the appropriate density in PBS. Bacteria were killed by heating suspensions at 60°C for 1 hour. Killing was confirmed by plating the suspensions on agar plates and incubating them at 37°C overnight.

### Experimental animals and design

The use of mice has been guided by strict adherence to the protocols (ID# 09-15-2010:SKG/YA; ID# 01-03-2011: SKG/RR), approved specifically for this study, by the Indiana State University Animal Care and Use Committee (IACUC). Female BALB/c mice (6–8 weeks of age) were used for all experiments. Pathogen-free BALB/c mice were bought from Harlan Sprague-Dawley (Indiana, USA), kept in quarantine for two weeks and then relocated to specific mouse facility that is routinely monitored by veterinarians, IACUC and USDA inspectors. These mice were bred and maintained at the animal care facility of Indiana State University.

5 mg of particulate SIS-hydrated (SIS-H) alone, obtained from Cook Biotech (Bloomington, IN) and 5×10^6^ CFU of HKSA alone or in combination with SIS-H, in a total volume of 500 µl was administered intra-peritoneally into the BALB/c mice. One group of mice received PBS only (control). All experiments were performed and repeated at least 3 times with 3–4 mice per test group. Peritoneal lavages were collected as described below, 24 hours later.

### Collection of peritoneal cells and lavages

Peritoneal exudates were harvested using 3 ml of PBS with 19 gauge needles. The collected samples were centrifuged at 500×g for 10 minutes at 4°C and the supernatants were used for cytokine and chemokine analysis. Peritoneal cells were washed twice with PBS and used for profiling inflammation-related gene expression.

### Determination of cytokines and chemokines secreted in the peritonea

Cytokines and chemokines in peritoneal fluids were assessed using the mouse inflammatory cytokine array kits (Ray Biotech Inc., GA, USA) following the manufacturer's instructions. Briefly, the cytokine array membranes provided were blocked with 2 ml of blocking buffer for 30 minutes and then incubated overnight with 1 ml of undiluted samples at 4°C. Samples were then decanted, and the membranes washed three times with wash buffer. Membranes were incubated with biotin-conjugated primary antibodies (1∶250 dilutions) at room temperature for 2 hours, then washed and exposed to horseradish peroxidase-conjugated streptavidin (1∶1000 dilution) for 1 hour. This was followed by treatment with 500 µl of peroxidase substrate for 2 minutes in the dark. The signal intensities were read by a chemiluminescence reader (Epi Chemi II Darkroom, UVP) and analyzed using the Ray Biotech cytokine expression analysis software. Positive and negative controls from six array spots were used to normalize the results and the net result for each spot was determined after subtraction of the background intensity. Data are expressed as the fold changes relative to the PBS control of each cytokine or chemokine protein detected using pooled peritoneal fluids of 3–4 mice per group. The experiment was repeated at least 3 times.

### Real time polymerase chain reaction for transcriptome profiling of inflammasome-responsive genes

Peritoneal exudate cells isolated from the control (PBS) and experimental groups were used to profile inflammasome-responsive gene expression by semi-quantitative RT-PCR. Briefly, total RNA was extracted from the peritoneal exudate cells according to the manufacturer's protocol (Ambion, Austin, TX). The quality of the RNA preparation was first assessed by spectrophotometry. All samples had 260/280 ratios above 2.0 and 230/260 ratios above 1.7. Further assessment was done using quality control plates (PAMM-999A-1, SA Biosciences, Frederick, MD). Then equal amounts of RNA (1 µg) from all samples were subjected to first-strand cDNA synthesis using RT2 first-strand kit from SA Biosciences, followed by PCR amplification. We used the RT2 Profiler PCR inflammasome array (PAMM-097) from SA Biosciences for the transcriptome analysis of inflammasome-associated genes. The experiments were performed in a Stratagene Mx3000P cycler using a cycling program provided by the manufacturer. Data were analyzed and fold changes in values were calculated using the PCR array analysis tool available at their website (http://pcrdata-analysis.sabiosciences.com/pcr/arrayanalysis.php). Gene expressions were normalized with respect to five house-keeping genes included in the array kit and expressed as averages of log2 ratios. Fold regulation changes of genes that differed by ≤ or ≥1.5 compared to PBS controls was considered significant.

### Statistical analysis

All data are expressed as mean ± SD. Data were analyzed by one-way analysis of variance (ANOVA) followed by a Tukey's post hoc test or the Student's *t*-test wherever relevant. For all analysis GraphPad Prism version 5 (GraphPad Software, San Diego, CA) was used. A *p*-value<0.05 was considered statistically significant.

## Results and Discussion

### Impact of HKSA with or without SIS-H on the Th1/Th2 cytokine profile

**Figure 1:**
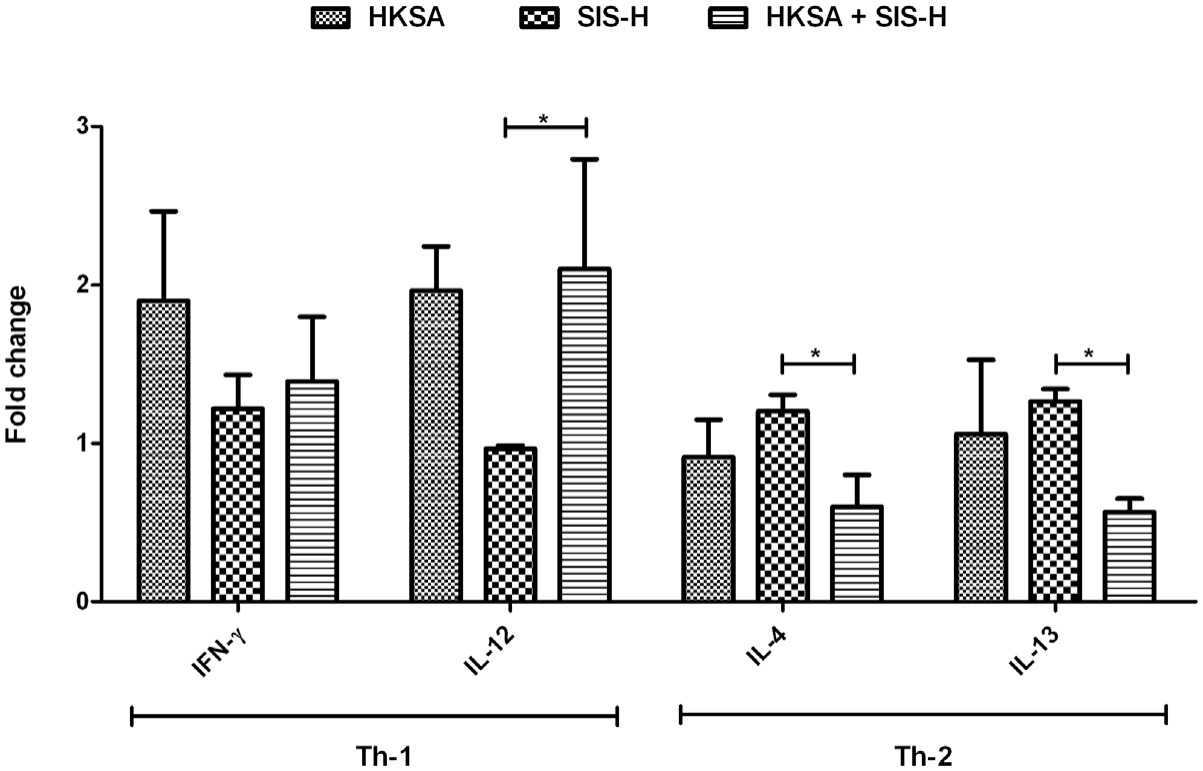
Shift in Th1-Th2 balance induced by the combined action of HKSA and SIS-H. HKSA in the presence of SIS-H shows higher expression levels of Th1-associated cytokines (IFN- γ and IL-12) than Th2-associated cytokines (IL-4 and IL-13) relative to SIS-H alone. HKSA by itself promotes a Th1-type immune response. Data for each group used in the figure are based on fold change values obtained by comparing to the PBS control group. (* = *p*<0.05).

**Figure 2:**
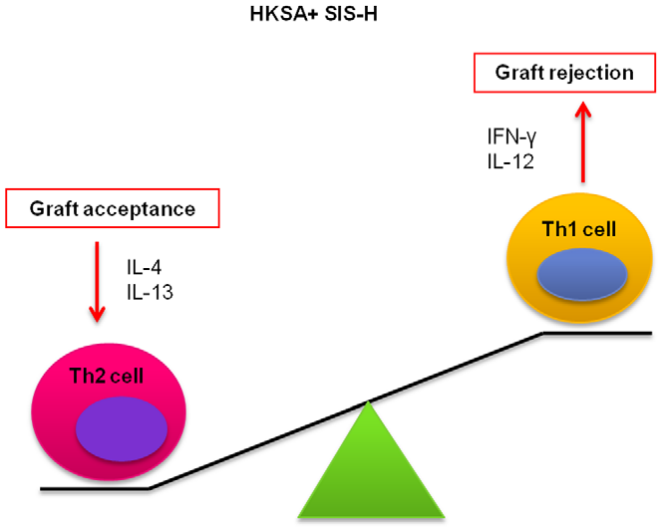
Diagrammatic representation of Th1/Th2 imbalance caused by HKSA-SIS-H exposure on graft transplantation. The addition of HKSA to SIS-H can promote graft rejection due to the higher expression levels of IFN-γ and IL-12 (Th1-type immune response) and lower expression levels of IL-4 and IL-13 (Th2-type immune response). The Th2 response is known to promote graft acceptance.

It is now well documented that SIS-H alone primarily induces a Th2 response [18], [38], a signature profile promoting its broad application in tissue remodeling. In this study, we wanted to delineate how this Th1/Th2 profile changes in the presence of HKSA. We therefore determined the expression patterns of some key cytokines corresponding to Th1 (IFN-γ and IL-12) and Th2 (IL-4 and IL-13)-mediated immune response, using the cytokine antibody-array technique. Our results showed that HKSA in the presence of SIS-H (or in other words, SIS-H contaminated with HKSA) induced higher levels of IFN-γ and significantly higher IL-12, but significantly lower levels of IL-4 and IL-13 as compared to SIS-H alone (Figure 1). HKSA by itself registered a Th1-biased immune response (IFN-γ = 1.9 fold and IL-12 = 1.963 fold as compared to IL-4 = 0.912 fold and IL-13 = 1.05 fold). Such a Th1-type immune profile of HKSA clearly suppressed the Th2 response mediated by SIS-H, while augmenting the Th1 immune response. These results demonstrated a shift in the T helper profile from type 2 to type 1 induced in response to the exposure of SIS-H to HKSA. This switch to the Th1 response could have important consequences such as delayed wound healing and aggravated graft rejections, as illustrated in Figure 2.

### Effects of HKSA-SIS-H exposure on the milieu of cytokines, chemokines and growth factors

**Figure 3:**
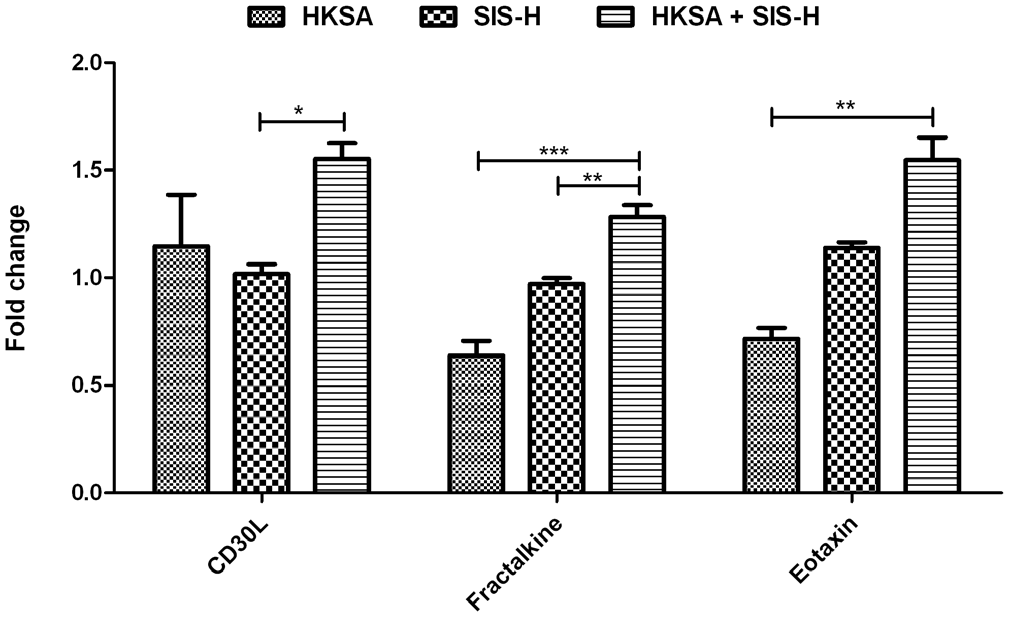
Effect of the combined action of HKSA and SIS-H on CD30L, Eotaxin and Fractalkine. The expression levels of CD30L, Eotaxin and Fractalkine are up-regulated due to the combined exposure of HKSA and SIS-H relative to SIS-H alone. All three of these molecules are known to promote graft rejections. Data for each group used in the figure are based on fold change values obtained by comparing to the PBS control group (* = *p*<0.05, ** = *p*<0.01 and *** = *p*<0.001).

Early inflammatory events play a critical role in shaping the outcome of the wound healing or tissue remodeling process [39]. The cytokine/chemokine cascades can drive the recruitment of various immune cells like neutrophils and macrophages that can either promote or aggravate wound healing. In this study, we examined the induction of some inflammatory cytokines and chemokines by SIS-H alone, and HKSA alone or in combination with SIS-H, and discussed their possible roles in tissue remodeling, graft-versus-host disease (GVHD) and wound healing. Our results showed that the addition of HKSA could impair the efficacy of SIS-H by promoting adverse inflammation and subsequent tissue damage. This is clearly evident in the augmented expressions of CD30L, Fractalkine and Eotaxin, relative to the levels induced by SIS-H alone (Figure 3). The addition of HKSA had a cumulative effect on the expression of these cytokines, particularly CD30L and Fractalkine. CD30L-CD30 signaling can promote CD4^+^ T-cell mediated GVHD [40], [41]. The increased expression of Fractalkine may also be unfavorable for the host, since the blocking of Fractalkine, also called MAdCAM-1, is known to alleviate graft-versus-host reaction [42]. Eotaxin is the primary chemoattractant for eosinophils and drives their maturation, migration and activation. Although acute graft rejections are primarily attributed to Th1 responses, Th2-biased eosinophilic inflammation can also mediate rejections. Eosinophils can promote detrimental tissue damage by the release of cationic granules and cytokines like IL-3 that stimulate further inflammation [43].

**Figure 4:**
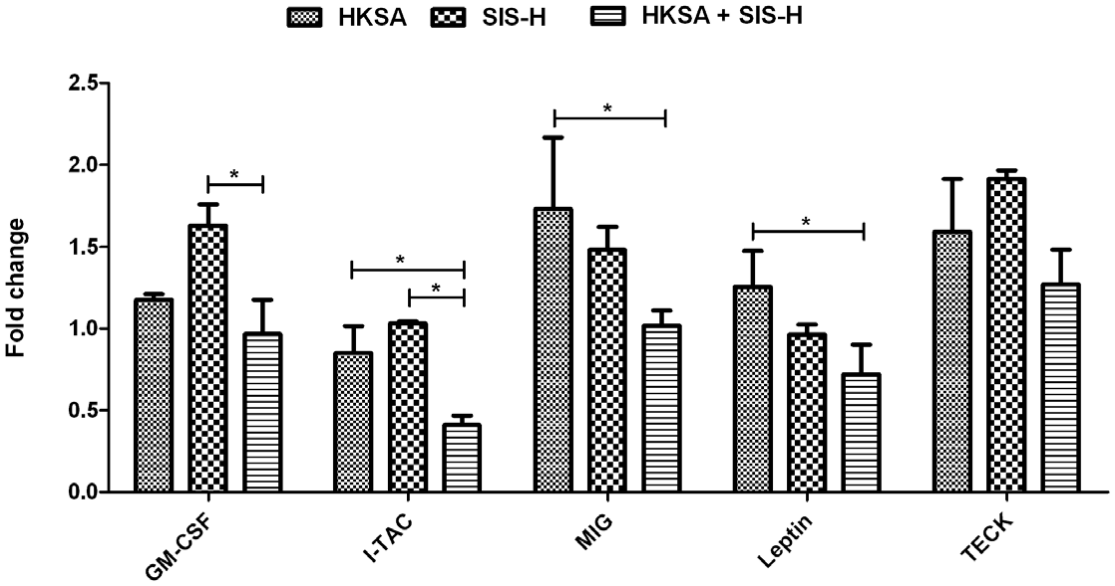
Down-regulation of inflammatory cytokines due to exposure to HKSA together with SIS-H. The growth factor GM-CSF, antibacterial chemokines I-TAC, MIG, TECK and adipokine Leptin are down-regulated in response to HKSA plus SIS-H. SIS-H by itself can induce high levels of these inflammatory molecules. Significant suppression of these molecules can impair bacterial clearance and delay wound healing. Data for each group used in the figure are based on fold change values obtained by comparing to the PBS control group. (* = *p*<0.05).

The augmented expression of CD30L, Fractalkine (a potent T-cell and monocyte chemoattractant) and Eotaxin due to the combined action of HKSA and SIS-H, compared to either HKSA or SIS-H alone, implies an increased mobilization and activation of leukocytes like eosinophils, monocytes, macrophages and mast cells that can lead to detrimental inflammatory response. However, the expression of a number of chemokines was found to be suppressed when SIS-H was combined or contaminated with HKSA. The chemokines modulated include GM-CSF, I-TAC, MIG, Leptin and TECK. The granulocyte-macrophage colony stimulating factor (GM-CSF) binds to receptors belonging to the gp140 family of proteins and stimulates hematopoiesis [44]. Since granulocytes play important defensive roles in the course of *S. aureus* infection [45], GM-CSF is vital in bacterial clearance. GM-CSF also plays a key role in the development of dendritic cells that are specialized in antigen uptake and presentation [46]. Furthermore, GM-CSF can be therapeutically useful during organ transplantation, as it does not induce graft rejection and help resist infections [47], [48]. Our findings showed that SIS-H by itself induced high expression of GM-CSF, which was significantly suppressed upon the addition of HKSA (Figure 4). This suppression of GM-CSF could be detrimental to the host in terms of bacterial clearance, innate-adaptive immune system crosstalk and graft rejections.

**Figure 5:**
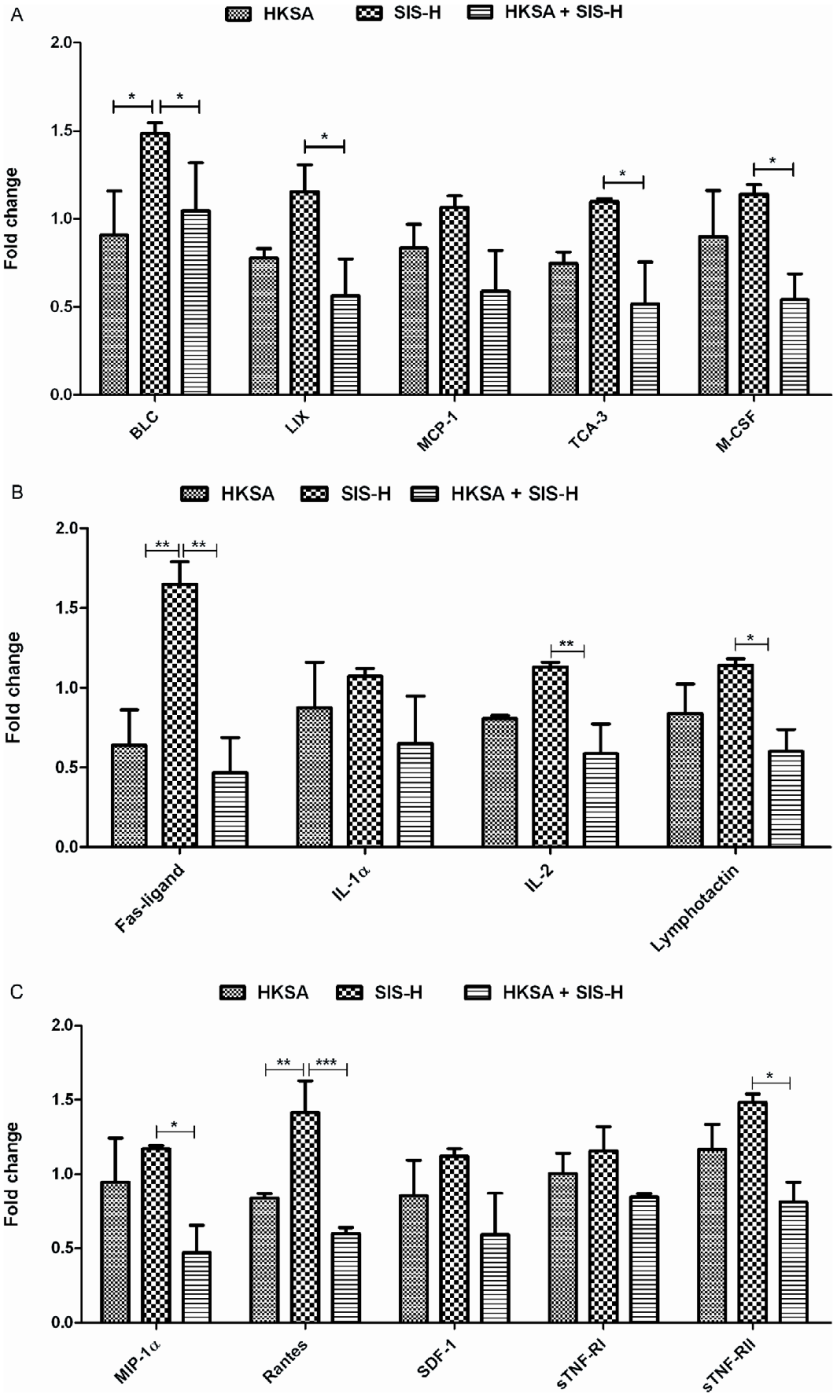
(A–C): Effect of HKSA-SIS-H exposure on the expression of inflammatory cytokines and chemokines involved in wound healing and GVHD. Data for each group used in the figure are based on fold change values obtained by comparing to the PBS control group. (* = *p*<0.05, ** = *p*<0.01 and *** = *p*<0.001).

The chemokines MIG (Monokine induced by IFN-γ or CXCL9) and I-TAC (IFN-γ-inducible T-cell chemoattractant or CXCL11) regulate lymphocyte mobilization [49] and have antimicrobial properties [50]. These CXCR3 chemokines have also been implicated in transplant rejections [51]. The expression of MIG and I-TAC were significantly decreased due to the combined HKSA-SIS-H exposure, relative to HKSA alone and SIS-H respectively (Figure 4). The other cytokines suppressed by the HKSA-SIS-H combination were Leptin and TECK (Figure 4). Leptin is an adipokine, which is known to influence both the innate and adaptive arms of immunity. It can stimulate monocyte proliferation and lymphocyte activation. It is also known to promote a Th1 type immune response [52]. TECK or thymus-expressed chemokine is an activator of dendritic cells and thymocytes [53] and promotes leukocyte migration or inflammation. It has been shown to play a critical role in wound healing [54]. Thus, any suppression of TECK, due to the HKSA-SIS-H exposure, may also negatively impact the wound healing process. In addition to these chemokines, we observed that SIS-H by itself induced enhanced expressions of BLC (B-lymphocyte chemoattractant), LIX (Lipopolysaccharide induced CXC chemokine), MCP-1 (monocyte chemotactic protein-1), TCA-3 (T-cell activation-3) and M-CSF (macrophage-colony stimulating factor) (Figure 5A). However, the addition of HKSA to SIS-H significantly down-regulated the expressions of BLC, LIX, TCA-3 and M-CSF (Figure 5A). M-CSF is important in wound repair [55] and its suppression could therefore impair the wound healing response. Similarly, TCA-3 (or CCL1) and MCP-1 also have multiple functions in the process of wound healing [56], [57].

Furthermore, a number of other inflammatory cytokines, such as Fas-ligand, IL-1α, IL-2, lymphotactin, MIP-1α, Rantes, SDF-1 and sTNFRs (I and II) that were down-regulated by the combined action of SIS-H and HKSA, compared to SIS-H or HKSA alone, are depicted in figures 5B and 5C. Fas-ligand, which is important for the host's ability to control GVHD [58], is significantly down-regulated by SIS-H plus HKSA, relative to SIS-H alone (Figure 5B). Like Fas-ligand, low-dose IL-2 has also been reported to be vital in the clinical management of GVHD for its ability to promote the growth and survival of T-regulatory cells [59]. Additionally, the chemokines MIP-1α, Rantes and SDF-1 that play critical roles in accelerating wound healing [60], [61], were highly suppressed in the presence of HKSA together with SIS-H, compared to SIS-H alone (Figure 5C). The expression of sTNFRs has been shown to suppress clinical manifestations of GVHD [62]. It is therefore clear from the above discussion that the contamination of SIS-H with HKSA could repress the expression of critical cytokines thereby impairing wound repair and exacerbating GVHD.

### Effects of HKSA-SIS-H exposure on inflammasome-responsive genes

The members of the Nod-like receptor (NLR) family can form multi-protein complexes called inflammasomes that regulate the secretion of pro-inflammatory cytokines like IL-1β [63]. IL-1β is a key mediator of inflammation and is known to be a major protective cytokine against *S. aureus* infection [64]. However, increased production of IL-1β can also aggravate GVHD pathophysiology [65]. Studies have identified different inflammasome pathways like Nlrp1, Nlrp3 and Ipaf. Apart from their role in infection, the inflammasomes have been linked to pyroptosis and autophagy [63]. Additionally, the Nlrp3 inflammasome is known to mediate the adjuvanticity of alum [66], [67]. The role of the inflammasome in wound healing is also becoming increasingly apparent [68]–[70].

Because of the involvement of the inflammasome in infection, adjuvanticity, wound healing and GVHD, we analyzed the influence of HKSA with or without SIS-H on the mRNA expression patterns of various inflammasome-responsive genes. We have previously reported that SIS-H by itself does not activate any of the known inflammasome pathways [18]. However, *S. aureus* has been reported to stimulate the Nlrp3-mediated inflammasome pathway [71]. We therefore wanted to determine if the presence of SIS-H could alter the inflammasome-responsive immune profile induced by HKSA. It is clear from our above discussions that HKSA greatly impacted the immune microenvironment induced by SIS-H. It was therefore of interest to see if SIS-H could impact the pathophysiology due to HKSA. This would be particularly important while evaluating the adjuvant properties of SIS. Inflammasome-responsive genes, like IFN-γ, IL-1β, Tnf and Irf-1 that were highly induced (fold regulation ≥1.5) by both HKSA alone and in combination with SIS-H are presented in Table 1. IFN-γ, IL-1β and Tnf are pro-inflammatory cytokines that indicate the Th1-biased immune response engendered by HKSA alone and in the presence of SIS-H. These results support our cytokine antibody array data. Genes that were significantly up-regulated due to the combined exposure of HKSA and SIS-H, relative to HKSA alone, are listed in Table 2. Among these genes are IL-12b, MyD88 and NLR family proteins, Nlrp5, Nlrp6, Nlrp9b and Nlrx1. The enhanced expression of IL-12, as mentioned before, indicates a Th1-type response that may promote graft rejections but also *S. aureus* clearance [72]. Nlrx1 is known to induce the production of reactive oxygen species [73] and this may aid in *S. aureus* killing. However, a number of inflammasome-related genes, like IL-6, IL-33, Cxcl1, Casp1 and Casp12 were significantly down-regulated by the combined action of HKSA and SIS-H, relative to HKSA alone (Table 3). The fact that the combined exposure of HKSA and SIS-H promotes a Th1-response is further supported by the suppression of the pro-inflammatory cytokines IL-33 and IL-6. Both these cytokines are known to promote Th2 and limit Th1 immune responses [74]–[76]. Their significant suppression therefore indicates the stimulation of a Th1-biased immune response, which could exacerbate GVHD. Additionally, IL-6 has been shown to exhibit protective roles against *S. aureus* infections [77], suggesting that reduced expression of IL-6 may impair bacterial clearance. Cxcl1 or KC is a potent chemoattractant for neutrophils. Since neutrophils are crucial in host defense against *S. aureus* [78] and also in the wound healing process [79], suppression of this chemokine might adversely affect infection control and the wound healing response.

**Table 1:** Genes highly expressed by HKSA alone and in combination with SIS-H.

Inflammasome-responsive genes	Fold-regulation	
	HKSA	HKSA+SIS-H
IFN-γ (Interferon-gamma)	2.229	1.918
IL-1β (Interleukin-beta)	1.523	1.525
Irf-1 (Interferon regulatory factor 1)	1.678	1.601
Tnf (Tumor-necrosis factor)	1.501	1.612

Fold regulation values ≥1.5 (compared to the PBS control) are considered significant.

**Table 2:** Genes significantly up-regulated by HKSA-SIS-H combination relative to HKSA alone.

Inflammasome-responsive genes	Fold-regulation		
	HKSA	HKSA+SIS-H	*P* value
IL-12b (Interleukin-12b)	1.773	1.980	0.0103
MyD88 (Myeloid differentiation primary response gene 88)	−1.006	1.243	0.0006
Nlrp5 (NLR family, pyrin domain containing 5)	−1.317	1.221	0.0005
Nlrp6 (NLR family, pyrin domain containing 6)	−1.218	1.200	0.0025
Nlrp9b (NLR family, pyrin domain containing 5)	1.077	2.014	0.0121
Nlrx1 (NLR family member X1)	−1.085	1.128	0.0015

*P*-values were calculated using the Student's *t*-test. *P*-value<0.05 was considered significant.

**Table 3:** Genes significantly down-regulated by HKSA-SIS-H combination relative to HKSA alone.

Inflammasome-responsive genes	Fold-regulation		
	HKSA	HKSA+SIS-H	*P* value
IL-6 (Interleukin-6)	1.129	−1.986	0.0024
IL-33 (Interleukin-33)	−1.048	−1.922	0.0130
Cxcl1(Chemokine (C-X-C motif) ligand 1)	−1.066	−1.773	0.0078
Casp1 (Caspase1)	1.314	1.006	0.0103
Casp12 ( Caspase12)	1.344	−1.083	0.0014

*P-*values were calculated using the Student's t-test. *P*-value<0.05 was considered significant.

Most of the inflammasome-associated genes like Aim2, Bcl2, Card6, Naip1, Naip5, Nlrp1a, Nlrp3, Nlrp4b, Nlrp4e, Ptgs2 and others, listed in Table 4, did not show significant change in their expression patterns when exposed to HKSA together with SIS-H, versus HKSA alone. It is noteworthy that neither HKSA alone, nor HKSA with SIS-H induced the activation of the Nlrp3 inflammasome after 24 hours. Interestingly, the other common inflammasome pathways like Nlrp1 and Aim2 were also not activated by HKSA in the presence or absence of SIS-H.

**Table 4:** Genes whose expressions do not change significantly due HKSA-SIS-H exposure.

Inflammasome-responsive genes	Fold-regulation	
	HKSA	HKSA+SIS-H
Aim2 (Absent in melanoma 2)	−1.618	−1.582
Bcl2 ( B-cell leukemia/lymphoma 2)	−1.896	−1.814
Birc3 (Baculoviral IAP repeat-containing 3)	−1.046	−1.138
Card 6 (Caspase recruitment domain family, member 6)	−1.818	−1.781
Chuk ((Conserved helix-loop-helix ubiquitous kinase)	−1.118	−1.309
Ctsb (Cathepsin B)	1.203	1.120
Ifnb1 (Interferon beta 1, fibroblast)	−1.398	−1.214
IL-18 (Interleukin-18)	1.025	1.009
Mapk 12 (Mitogen-activated protein kinase 12)	−1.952	−1.767
Mapk 13 (Mitogen-activated protein kinase 12)	−1.198	−1.313
Mefv (Mediterranean fever)	1.398	1.251
Naip1 (NLR family, apoptosis inhibitory protein 1)	−1.785	−1.566
Naip5 (NLR family, apoptosis inhibitory protein 5)	−1.222	−1.384
Nfkb1(Nuclear factor of kappa gene enhancer in B-cells 1)	1.297	−1.301
Nlrp1a (NLR family, pyrin domain containing 1A)	−1.056	−1.012
Nlrp3 (NLR family, pyrin domain containing 3)	−1.076	−1.197
Nlrp4e (NLR family, pyrin domain containing 4e)	−1.629	−1.511
Nod2 (Nucleotide-binding oligomerization domain containing 2)	−1.096	−1.157
Panx1 (Pannexin 1)	−1.460	−1.317
Ptgs2 (Prostaglandin-endoperoxide synthase 2)	1.185	1.267
Pycard (PYD and CARD domain containing)	−1.256	−1.326
Rela (V-rel reticuloendotheliosis viral oncogene homolog A)	−1.109	−1.171
Tirap (Toll-interleukin 1 receptor (TIR) domain-containing adaptor protein)	−1.091	−1.303
Txnip (Thioredoxin interacting protein)	−1.191	−1.228

**Figure 6:**
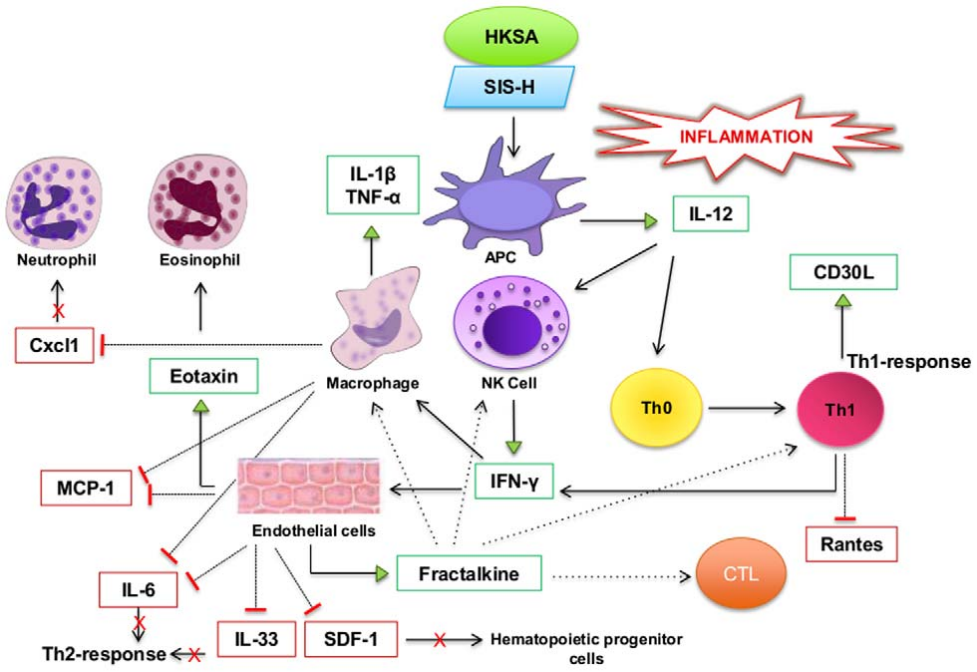
A simplistic model of the local microenvironment (cytokines and infiltrating cells) induced by the combined action of HKSA and SIS-H. The uptake of HKSA and SIS-H by APCs can release IL-12, which can stimulate NK cells and T-cells to produce IFN-γ. The IFN-γ can in turn activate macrophages to produce pro-inflammatory cytokines IL-1β and Tnf-α and endothelial cells to produce the chemokines Eotaxin and Fractalkine. Eosinophil is a potent chemoattractant of eosinophils and the induced Fractalkine can cause chemotaxis of NK cells, macrophages, CD4^+^ and CD8^+^ T-cells. The Th1-cells can CD30L, which can influence both the innate and acquired arms of immunity. Such an immune microenvironment can cause heightened local inflammation.  = Suppression,  = Activation.

It is therefore clear from our cytokine-chemokine and inflammasome array studies that the combined action of HKSA and SIS-H evokes a unique local immune microenvironment that is distinct from those induced by either HKSA or SIS-H alone. Based on the cytokine-chemokine and inflammation-responsive gene array data, the picture that emerges of the immune microenvironment in BALB/c mice from the combined action of HKSA and SIS-H has been depicted in Figure 6.

## Conclusion

Our studies revealed that *S. aureus* can severely compromise the efficacy of SIS as a biomaterial, primarily because of its impact on the immune microenvironment surrounding the affected host tissues. *S. aureus* in conjunction with SIS promotes a Th1-biased immune response (IL-12, IFN-γ), suppresses the Th2 cytokine profile (IL-4, IL-13, IL-33 and IL-6), induces the expression of pro-inflammatory cytokines like IL-1β and Tnf and down regulates the expression of a number of inflammasome-responsive genes, Nlrp3, Aim2 etc. Other important pro-inflammatory molecules such as CD30L, Fractalkine and Eotaxin are also augmented from the *S. aureus*-SIS exposure. A variety of chemokines and growth factors like GM-CSF, Cxcl1, MIG, I-TAC and Leptin are also modulated. Such an immune proteome implying heightened inflammation and Th1 response can aggravate graft survival, hinder bacterial clearance and therefore be detrimental to the host. Overall, the information gained about the modulation of the host innate immune microenvironment by the *S. aureus*-SIS exposure will help define new therapeutic targets for developing effective intervention strategies for wound-related infections.

SIS is an FDA approved [1] and highly biocompatible tissue scaffold material that is also an effective immunoadjuvant. However, one of the major concerns associated with SIS has been surgical site infection and wound contamination. A number of studies have therefore focused on improving the anti-bacterial property of SIS by incorporating molecules like silver nanoparticles [80] and bismuth thiol [81]. However, the first step to finding a potential intervention to this clinical problem should be the analysis of the complex host-SIS-bacteria interactome. The issue for future studies would be to determine if the adversarial immune microenvironment engendered due to contamination by *S. aureus* or other bacterial products could be mitigated by including antibacterial compounds in SIS biomaterials.
